# Post-weaning stroking stimuli induce affiliative behavior toward humans and influence brain activity in female rats

**DOI:** 10.1038/s41598-021-83314-w

**Published:** 2021-02-15

**Authors:** Shota Okabe, Yuki Takayanagi, Masahide Yoshida, Tatsushi Onaka

**Affiliations:** Division of Brain and Neurophysiology, Department of Physiology, Jichi Medical University, 3311-1 Yakushiji, Shimotsuke-shi, Tochigi-ken, 329-0498 Japan

**Keywords:** Neuroscience, Physiology

## Abstract

Gentle touch contributes to affiliative interactions. We investigated the effects of gentle stroking in female rats on the development of affiliative behaviors toward humans and we exploratively examined brain regions in which activity was influenced by stroking. Rats that had received stroking stimuli repeatedly after weaning emitted 50-kHz calls, an index of positive emotion, and showed affiliative behaviors toward the experimenter. Hypothalamic paraventricular oxytocin neurons were activated in the rats after stroking. The septohypothalamic nucleus (SHy) in the post-weaningly stroked rats showed decreased activity in response to stroking stimuli compared with that in the non-stroked control group. There were negative correlations of neural activity in hypothalamic regions including the SHy with the number of 50-kHz calls. These findings revealed that post-weaning stroking induces an affiliative relationship between female rats and humans, possibly via activation of oxytocin neurons and suppression of the activity of hypothalamic neurons.

## Introduction

Mammals develop affiliative relationships with intraspecific individuals such as mother-infant relationships^1,2^ and male–female relationships^3,4^. They also show affiliative behaviors toward hetero-specific animals including humans^5–15^. Affiliative interactions with humans have positive effects on human health^16,17^ as well as on animal welfare^18,19^. Therefore, elucidation of the mechanisms of affiliative relationships between humans and animals may contribute to improvements in the health, welfare, and quality of life of humans and animals. However, little is known about the neural mechanisms underlying the development and maintenance of human-animal affiliative relationships.

Rats have been shown to emit two broad subgroups of ultrasonic vocalizations (USVs) depending on their emotional states; one is USV with a frequency range of 18–32 kHz, referred to as "22-kHz calls"^20–24^, which are emitted in aversive states, and the other is USV with higher frequency (more than 35 kHz), referred to as "50-kHz calls"^23–25^, which are emitted in appetitive or rewarding states. Among the 50-kHz calls, frequency-modulated calls (FM-calls), in particular, have been suggested to be an indicator of strong pleasurable emotion^26,27^. These characteristics make USVs useful for monitoring internal affective states of rats toward humans in experimental situations.

We previously found that gentle tactile stimulation during a post-weaning period induced emission of 50-kHz calls and resulted in the development of intimate relationships between humans and male rats possibly via activation of hypothalamic oxytocin neurons^28^. It has been suggested that gently tactile stimulation plays a fundamental role in the establishment and maintenance of intimate relationships between individuals^29,30^. In addition, oxytocin has been shown to facilitate affiliative social behaviors^31–34^, and massage or non-noxious tactile stimulation increases oxytocin release in animals^35–37^ and humans^38^.

Sex differences have been reported in emotional reactivity and sociability. For example, female rats show higher neuroendocrine responses to stressful stimuli than do male rats^39^ and have greater vulnerability to affective disease. On the other hand, male rats show playing behavior more frequently than female rats do^40,41^. Sexual dimorphism has also been demonstrated in the emission of USVs. Female rats vocalize 22-kHz calls more frequently than do male rats in response to stressful stimuli such as exposure to natural enemy scents^42^ but emit 22-kHz calls less frequently than do male rats in response to air-puff^43^. On the other hand, female rats vocalize 50-kHz calls less frequently than male rats do in response to tickling stimuli given by humans^25^. It has remained to be clarified whether female rats show the development of affiliative behavior toward humans and emit 50-kHz USVs in response to stroking stimuli as male rats do.

By using the post-weaning stroking procedure that we have established for inducing affiliative behaviors toward humans in male rats, the present study was carried out with the aim of clarifying affiliative responses in female rats toward humans. We found that female rats developed affiliative responsiveness toward humans. We also identified brain regions in which activities were correlated with the number of 50-kHz USVs emitted in response to stroking stimuli.

## Materials and methods

All protocols used in the present study were the same as those used in experiments with male rats as described previously^28^. The original source of method descriptions is our previous study^28^.

### Subjects

Animal experiments were conducted under approval from the Animal Experiment Committee of Jichi Medical University and were in accordance with the Institutional Regulations for Animal Experiments and Fundamental Guidelines for Proper Conduct of Animal Experiments and Related Activities in Academic Research Institutions under the jurisdiction of the Ministry of Education, Culture, Sports, Science and Technology. Animal experiments were performed in accordance with the “Animal Research: Reporting of In Vivo Experiments” (ARRIVE) guidelines (https://www.nc3rs.org.uk/arrive-guidelines).

Forty-two female Lewis rats were used in this study. These animals were produced in our laboratory by mating of rats obtained from a supplier (LEW/ CrlCrlj, Charles River Laboratories Japan, Inc., Kanagawa, Japan)^28^. After weaning at the age of three weeks, female rats were housed in pairs at controlled temperature (22 ± 2 °C) and humidity (55 ± 15%) under a 12-h light/dark cycle (lights on at 7:30 am to 7:30 pm). Food and water were available ad libitum.

### Groups and general experimental design

**Figure 1:**
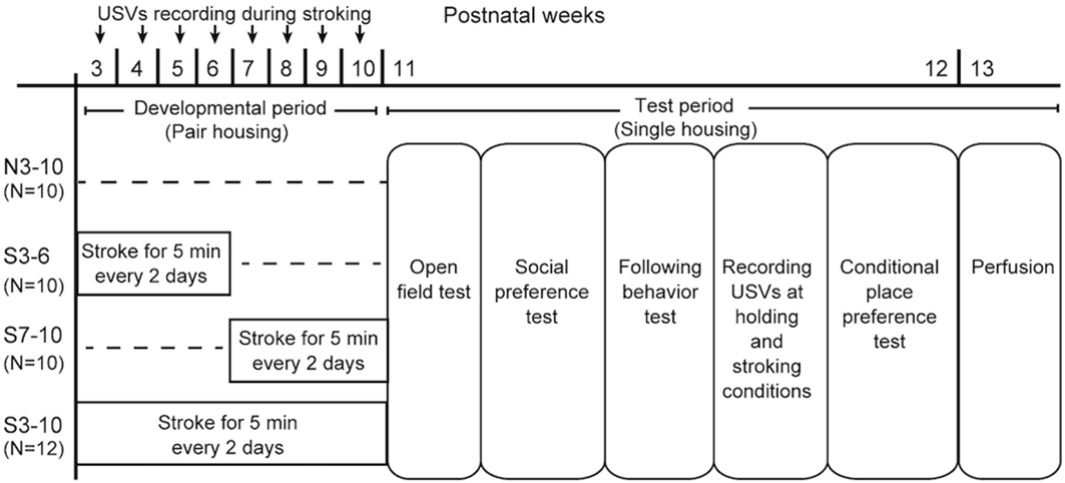
Time schedules of experiments. Ultrasonic vocalizations were recorded 5 min once per week between 3 and 10 weeks of age (developmental period) in the S3-6, S7-10, S3-10 groups. Behavioral tests were subsequently conducted in succession at the ages of 11 and 12 weeks (test period).

An experimenter (S.O.) gave stroking stimuli with the hand over the back of each rat at a speed of 5–10 cm/sec for 5 min every other day, as described previously^35^. Stroking stimuli by this procedure induced affiliative responsiveness toward humans in male rats in a previous study^35^. Female rats start to show a regular estrous cycle at approximately 6–7 weeks of age^44^. Estrogens affect social behaviors such as social recognition and aggressive behavior^45,46^. Thus, we chose periods before and after sexual maturation. 42 rats were divided into four groups to investigate the critical period for the development of affiliative behavior: S3-6 group, S7-10 group, S3-10 group, and N3-10 group. Rats in the S3-6 group received 5-min stroking stimuli from the experimenter for 4 weeks between 3 and 6 weeks of age (n = 10). Animals in the S7-10 group received stroking stimuli for 4 weeks between 7 and 10 weeks of age (n = 10). Animals in the S3-10 group received stroking stimuli for 8 weeks between 3 and 10 weeks of age (n = 12). Control rats in the N3-10 group did not receive stroking stimuli (n = 10). At 10 weeks of age, all of the rats were housed individually, and then behavioral tests were conducted. Time schedules of treatments and behavioral tests are shown in Fig. 1.

### Ultrasonic vocalizations

#### General protocols for recording

Ultrasonic vocalizations (USVs) were recorded using a microphone (Type 4158 N, Aco, Tokyo, Japan) designed for measurements of sound pressure levels of 20–100,000 Hz sounds, as described before^28^. The microphone was set at a distance of approximately 20 cm from a rat and was connected to a sensor amplifier (SR-2200, Ono Sokki, Kanagawa, Japan). Acoustic data were sampled at a rate of 250 kHz with 16 bits by an Avisoft RECORDER (Version 4.2, Avisoft Bioacoustics, Berlin, Germany). Recorded USVs were analyzed by using a software program of Avisoft SASLab Pro (Version 5.2, Avisoft Bioacoustics). Spectrograms were generated with a fast Fourier transform length of 256 points and an overlap of 75%.

#### Recording of USVs

In the S3-6, S7-10, and S3-10 groups, USVs were recorded during a 5-min stroking session once per week during the developmental period (Fig. 1). Each rat was placed on the experimenter’s lap and was given massage-like stroking stimuli with the hand of the experimenter (S.O.). The numbers of 50-kHz calls (frequencies between 35 and 100 kHz) and 22-kHz calls (frequencies between 18 and 32 kHz) were manually counted.

During the test period, USVs in the basal condition were recorded for 5 min when rats were kept in their individual home cages. Microphones were set above mesh lids of their home cages more than 25 min before recording. USVs in a holding condition were recorded for 5 min when each rat was placed on the experimenter (S.O.)’s lap. USVs in a stroking condition were then recorded during the next 5-min stroking period. The numbers of 50-kHz calls and 22-kHz calls were manually counted. Fifty-kHz calls were divided into FM calls and non-FM calls according to the shape of a spectrogram plot of each syllable as described previously^26^. The numbers of these types of USVs were counted in basal, holding, and stroking conditions.

### Behavioral tests

#### Open field test

During domestication processes of wild animals, development of affiliative behavior toward humans has often been associated with reduced levels of anxiety^47,48^. Thus, anxiety-related behavior was assessed by an open field test in this study. Each animal was placed in an open field (W60 × D60 × H40 cm) and kept there for 10 min. Behaviors were recorded by a video camera installed on the ceiling of the room. Total movement distance, total time spent staying in the center area, which was 36% of the total open field arena, and total time spent immobile were automatically measured by use of a behavior analyzing program, Time OFCR1 (O’Hara & CO., LTD, Tokyo, Japan).

#### Social preference test

Preference to the experimenter (S.O.)’s hand as compared to a novel object was assessed by a social preference test, as described before^28^. The experimenter (S.O.)’s hand was placed at one corner of a test box (W60 × D60 × H40 cm). At the diagonally opposite corner of the test box, a novel plastic bottle (W6 × D6 × H14 cm) was placed. Each animal was placed into the test box at a corner other than the corners where the hand and the bottle were placed. The time spent staying in a square (20 × 20 cm) of the hand or the bottle was measured automatically during a 10-min test period by use of a program (Time OFCR1). Preference score was calculated as [(time spent staying in the hand area) – (time spent staying in the bottle area)].

#### Following behavior test

Proactive approach behavior toward the experimenter (S.O.)’s hand (“following” behavior) was observed in a test box (W60 × D60 × H40 cm), as described before^28^. The experimenter (S.O.)’s hand was placed at a corner of the test box. Each animal was introduced at the corner diagonally opposite to the hand. The hand was moved from one corner to the next corner along the wall over a period of 3 s (20 cm/sec) and then kept at the corner for 7 s. This movement was repeated for 5 min. The time spent following the experimenter’s hand within a distance of approximately 3 cm was measured manually. In addition, the number of 50-kHz calls during the following behavior test was counted. The experiment and analysis were conducted blind to treatments of the animals.

#### Conditional place preference (CPP) test

The reward value of stroking stimuli was evaluated by a CPP test, as described before^28^. The test apparatus consisted of a black box (W40 × D30 × H30 cm) and a white box (W40 × D30 × H30 cm), which were adjacent with a boundary wall (30 × 30 cm). The boundary wall had a door hole (8 × 8 cm) through which rats could enter either box. The black box had black walls with a meshed floor and luminance was set to 10 lx. The white box had white walls and a grid floor, with the grids arranged parallel to the boundary wall, and there was no illumination. Rat behaviors were recorded by video cameras installed on the ceilings of both boxes. On day 1, each rat was placed in the white box and kept in the CPP test box for 10 min with the boundary door open so that the rat could enter either box. On day 2, each rat was placed in the black box with the door open for 10 min and then the time spent staying in each box was measured automatically by using a software program (Time LD4, O’Hara and Co.) to determine initial preference (pre-conditioning). The box in which each rat stayed longer was defined as the initially preferred box (I.P. box) and the other box was defined as the initially non-preferred box (I.N.P. box). Conditioning was performed during the period between day 3 and day 8 with the door connecting the two boxes being closed. On day 3, rats were placed in the I.P. box and kept there for 5 min. On day 4, rats were placed in the I.N.P. box and stroking stimuli were applied by the experimenter (S.O.) for 5 min. The 2-day conditioning procedures were repeated another 2 times. In total, conditioning was conducted 3 times from day 3 to day 8. On day 9, rats were placed in the I.P. box with the door open and the time spent in the I.N.P. box and locomotion distance in the I.N.P. box were measured by using a software program. Preference ratios were calculated as [(time spent staying in the I.N.P. box during the post-conditioning) / (time spent staying in the I.N.P. box during the pre-conditioning)] and [(locomotion distance in the I.N.P. box during the post-conditioning) / (locomotion distance in the I.N.P. box during the pre-conditioning)].

### Immunohistochemical detection of c-Fos protein in oxytocin-immunoreactive (-ir) neurons

The rats in each group were assigned to one of two conditions: a stroking condition and a non-touch control condition. In the stroking condition, rats received stroking stimuli for 5 min and were then placed back in their individual home cages. USVs were recorded during the stroking period. In the non-touch condition, rats were kept in their individual home cages. Ninety minutes after termination of stroking stimuli, the animals were deeply anesthetized with Avertin (tribromoethanol; 200 mg/kg, i.p.) and perfused transcardially with heparinized saline (20 U/mL) followed by 4% paraformaldehyde in 0.1 M phosphate buffer (pH 7.4) for 15 min. The brains were immediately removed from the skulls, post-fixed in 4% paraformaldehyde overnight, placed in 30% sucrose in 0.1 M phosphate buffer until they sank, and frozen in dry ice. The hypothalamic part of each frozen brain was sectioned coronally at 40 µm and processed for immunochemical detection of c-Fos protein and oxytocin as described previously^35^. Sections containing the dorsal zone of the medial parvicellular part of the caudal PVN (3 sections, from 1.92 mm to 2.24 mm posterior to the bregma), rostral PVN (6 sections, from 1.0 mm to 1.80 mm posterior to the bregma), supraoptic nuclei (SON, 7 sections, from 0.6 mm to 1.56 mm posterior to the bregma) or BNST (4 sections, from 0.72 mm to 1.20 mm posterior to the bregma) were examined at an interval of 160 µm in each rat. The sum of the numbers of c-Fos-ir neurons, oxytocin-ir neurons, and double-positive neurons was counted in each brain region, the boundary of which was determined according to a rat brain map^49^. The percentages of oxytocin-ir neurons expressing c-Fos protein were calculated. Brain sections were mounted on slide glasses without description of experimental treatments. No blind operation was performed.

### Immunohistochemical detection of c-Fos protein in the forebrain and midbrain

To identify brain regions that showed increased or suppressed activity, we examined the expression of c-Fos protein in various regions of the forebrain and midbrain of each of the rats in the N3-10 and S3-10 groups. Brain sections of 40 µm in thickness were prepared and processed for immunochemical detection of c-Fos protein. All images of sections processed for c-Fos protein detection were taken by a microscope and processed with an open-source image processing software ICY spot detector^50^. We analyzed 76 brain regions in the forebrain and midbrain. Each brain region of interest (ROI) was circumscribed in multiple sections according to a rat brain map^49^. The size and number of sections in each ROI were assigned to be the same among animals. The number of c-Fos-ir cells within each ROI was automatically counted. Detailed information on the ROI and number of sections for analysis is provided in the supplementary information (see supplementary Tables 1–3 and supplementary Fig. 1).

### Examination of the estrous cycle

Vaginal smears were obtained immediately before perfusion to check the estrus stage of the animals. The estrous cycle was classified as proestrus, estrus, metestrus, and diestrus.

### Statistical analysis

**Figure 2:**
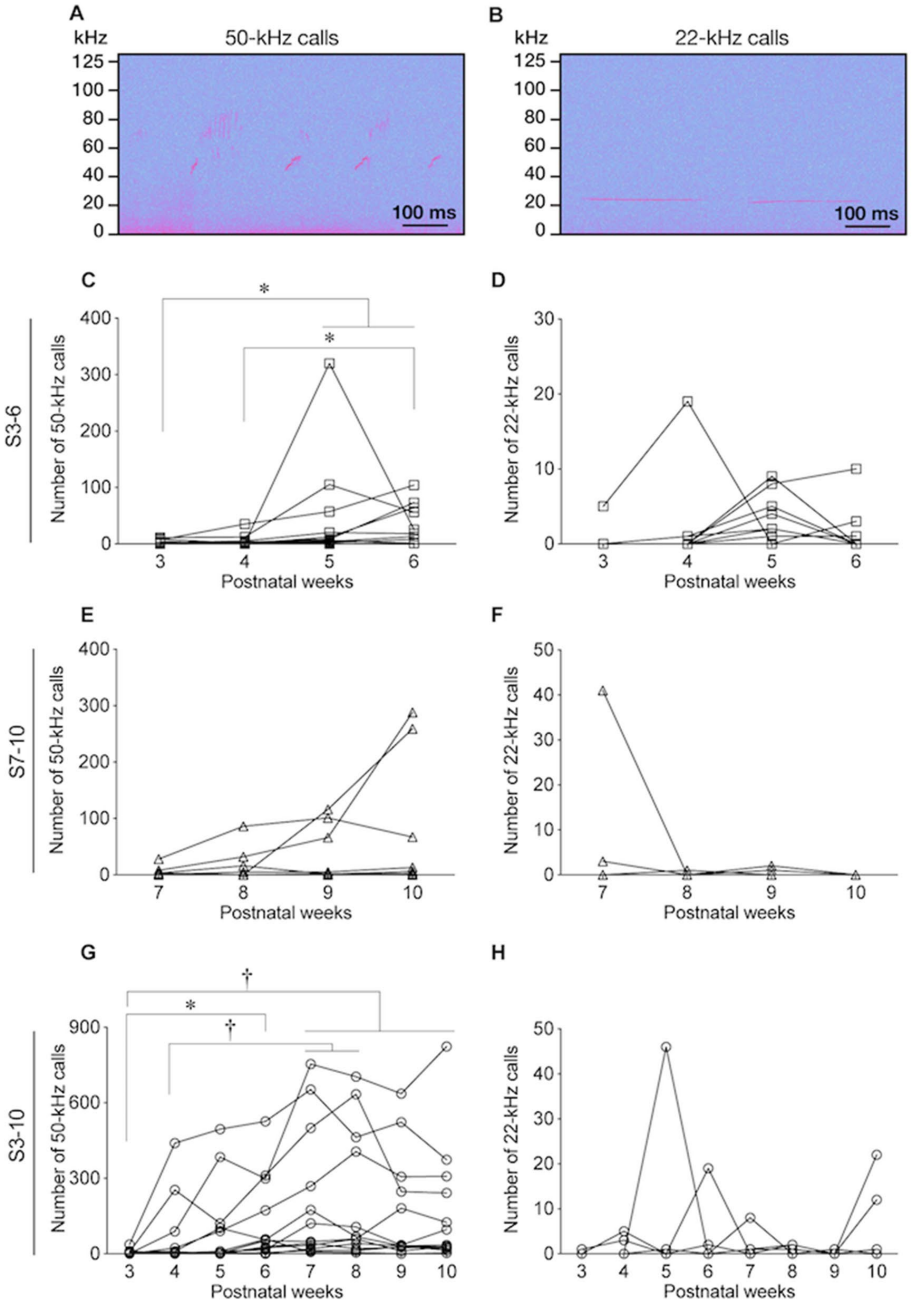
Ultrasonic vocalizations (USVs) during stroking stimuli. (**A**,**B**) Examples of spectrograms of 50-kHz calls (**A**) and 22-kHz calls (**B**). (**C**–**H**) Time courses of the numbers of vocalizations during stroking in the S3-6 group (**C**, 50-kHz; D, 22-kHz), S7-10 group (**E**, 50-kHz; F, 22-kHz), and S3-10 group (**G**, 50-kHz; H, 22-kHz). In the S3-6 group, the numbers of 50-kHz calls at the ages of 5 and 6 were significantly larger than the number of 50-kHz calls at the age of 3 weeks. In addition, the number of 50-kHz calls at the age of 6 weeks was significantly larger than that at the age of 4 weeks. In the S7-10 group, the number of 50-kHz calls did not significantly change. In the S3-10 group, the numbers of 50-kHz calls at 6, 7, 8, 9, and 10 weeks of age were significantly increased compared to the number at the age of 3 weeks. The numbers of 50-kHz calls at 7 and 8 weeks of age were significantly larger than the number at 4 weeks of age. The numbers of 22-kHz calls during stroking did not significantly change in any of the three groups. †, *P* < 0.01; *, *P* < 0.05, post-hoc Holm’s test.

**Figure 3:**
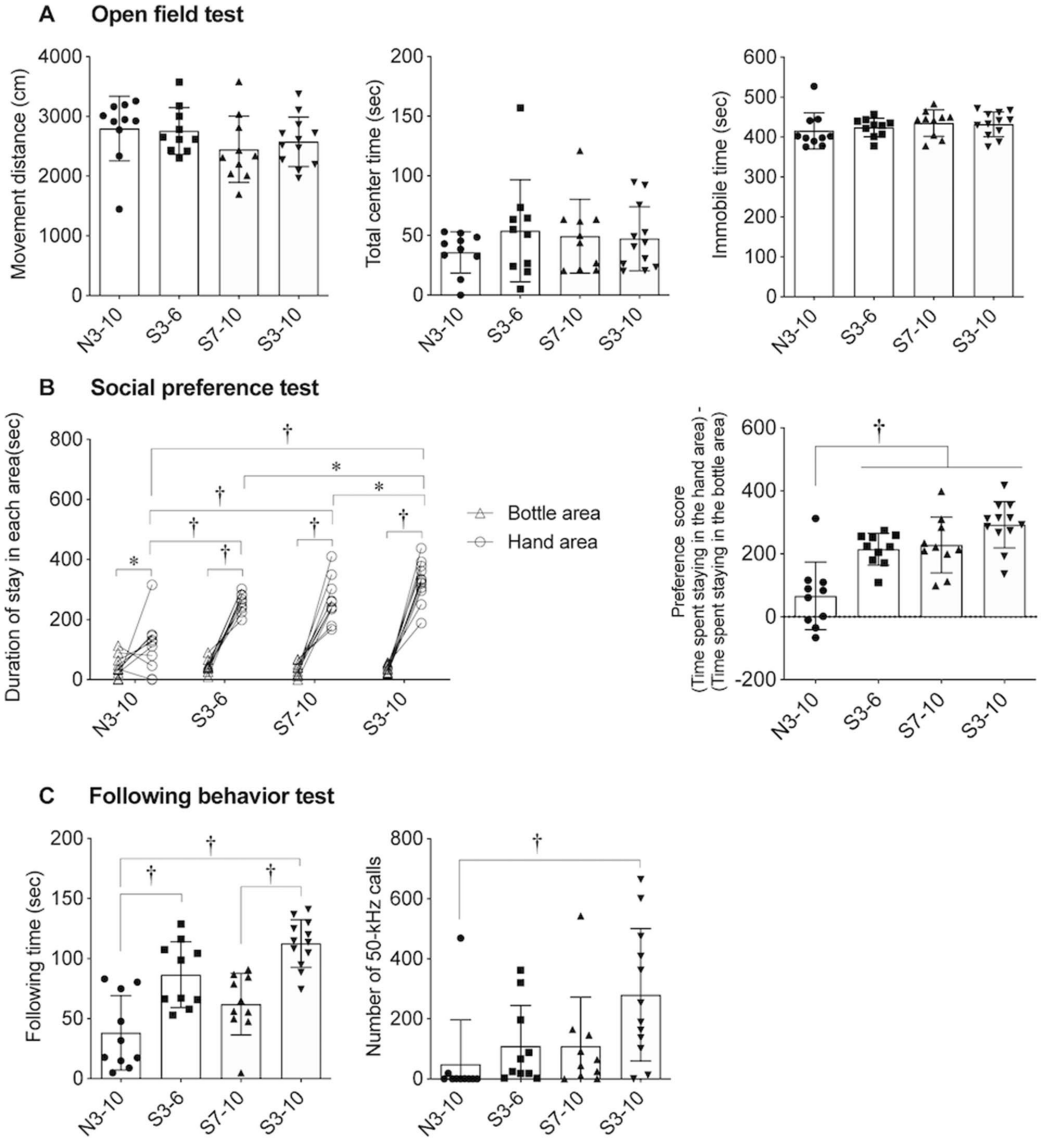
Results of the open field test, social preference test, and following behavior test. (**A**) Total distance of movements (left panel), total time spent staying in the center area (middle panel), and total time spent immobile (right panel) in an open field test. There were no significant differences in the results among the groups. (**B**) Durations and difference between durations of staying in the hand area and the bottle area in a social preference test. The times spent staying in the hand area in the S3-6, S7-10, and S3-10 groups were significantly longer than the time in the N3-10 group. In addition, the time spent staying in the hand area in the S3-10 group was significantly longer than the times in the S3-6, and S7-10 groups. Preference scores [(time spent staying in the hand area) – (time spent staying in the bottle area)] in the S3-6, S7-10, and S3-10 groups were significantly higher than the score in the N3-10 group. (**C**) Durations of following behavior and number of 50-kHz calls during the following behavior test. Durations of the following behavior (left panel) were significantly longer in the S3-6 and S3-10 groups than in the N3-10 group. In addition, duration of the following behavior was significantly longer in the S3-10 group than in the S7-10 group. The number of 50-kHz calls (right panel) was significantly larger in the S3-10 group than in the N3-10 group. †, *P* < 0.01; *, *P* < 0.05. One-way ANOVA followed by post-hoc Holm’s test (**A** and **C**, left) and repeated measures two-way ANOVA (group × area) followed by post-hoc Holm’s test (**B**) were performed. The Kruskal–Wallis test followed by post-hoc Holm’s test was also performed (**C**, right). Error bars denote standard error of the mean.

**Figure 4:**
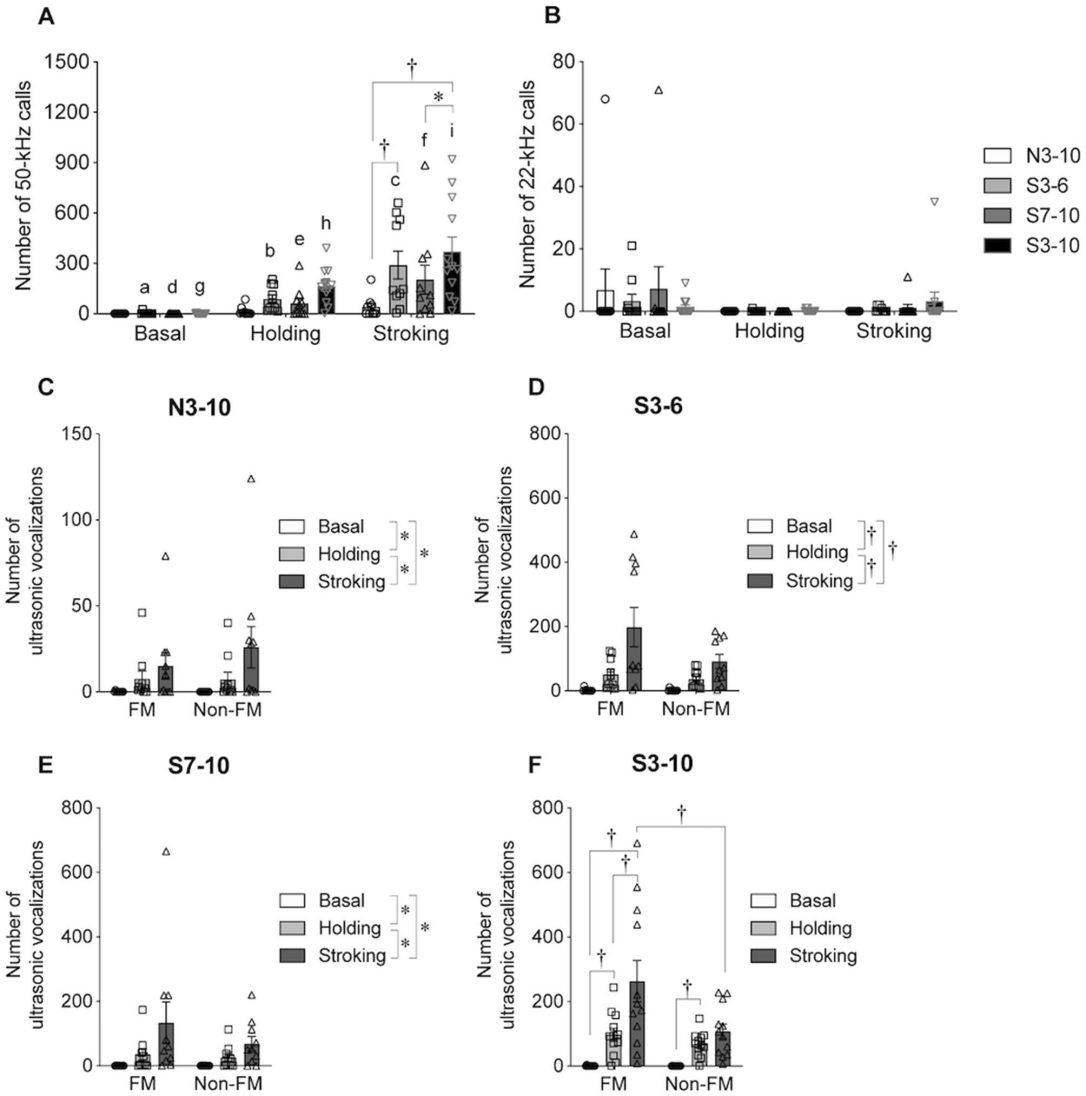
Numbers of ultrasonic vocalizations. (**A**) Number of 50-kHz calls. (**B**) Number of 22-kHz calls. The numbers of 50-kHz calls were significantly larger in the stroking condition than in the basal and holding conditions in the S3-6, S7-10, and S3-10 groups. In addition, in the S3-6, S7-10, and S3-10 groups, the numbers of 50-kHz calls were significantly larger in the holding condition than in the basal condition. In a group comparison, the numbers of 50-kHz calls in the stroking condition in the S3-6 and S3-10 groups were significantly larger than the number in the N3-10 group. The number of 50-kHz calls in the stroking condition in the S3-10 group was significantly larger than that in the S7-10 group. The numbers of 22-kHz calls were not significantly different in the basal, holding, and stroking conditions. (**C**–**F**) Numbers of FM and non-FM calls in the N3-10 (**C**), S3-6 (**D**), S7-10 (**E**), and S3-10 (**F**) groups. In the N3-10, S3-6, and S7-10 groups, the numbers of calls in the stroking condition were significantly larger than those in the basal or holding condition. The number of calls in the holding condition was significantly larger than that in the basal condition. In the S3-10 group, the number of FM calls in the stroking condition was significantly larger than those in the basal and holding conditions. In addition, the number of FM calls was significantly larger than the number of non-FM calls in the stroking condition. The number of non-FM calls in the holding condition was significantly larger than that in the basal condition. †, *P* < 0.01; *, *P* < 0.05. a vs. b, *P* < 0.01; a vs. c, *P* < 0.01; b vs. c, *P* < 0.01; d vs. e, *P* < 0.05; d vs. f, *P* < 0.05; e vs. f, *P* < 0.05; g vs. h, *P* < 0.01; g vs. i, *P* < 0.01; h vs. i, *P* < 0.01. A comparison was conducted by repeated measures two-way ANOVA (group × condition) or (syllable pattern × condition) followed by post-hoc Holm’s test. Error bars denote standard error of the mean.

**Figure 5:**
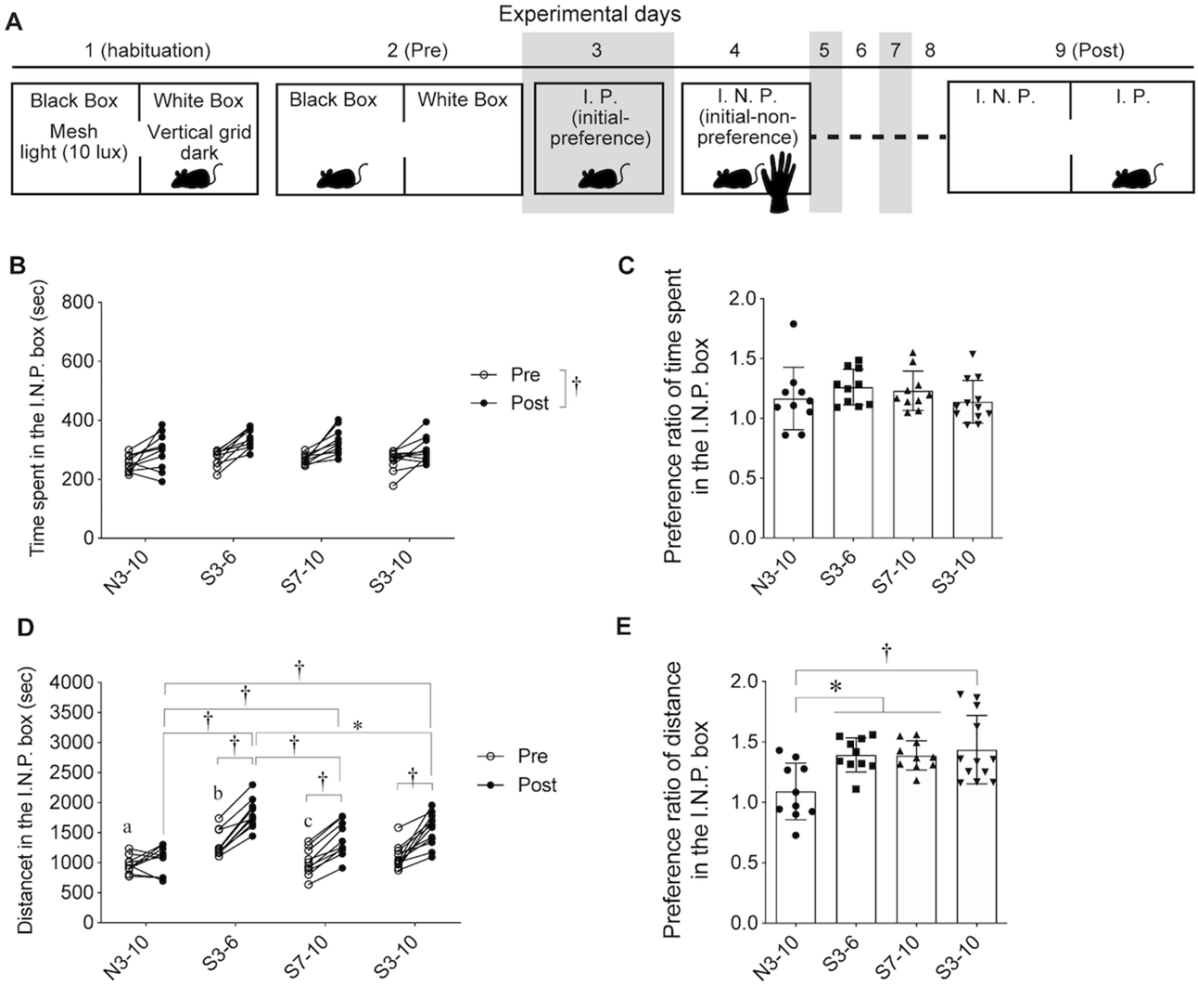
Results of the conditional place preference test. (**A**) Time course of experimental procedures for the conditioned place preference test. (**B**) Times spent staying in an initial non-preference (I.N.P.) box before (pre) and after (post) conditioning. Time spent staying in an I.N.P. box where rats received stroking stimuli was significantly longer after conditioning than that before conditioning. (**C**) Preference ratio of time spent in the I.N.P. box. The preference ratio of time [(time spent staying in the I.N.P. box during the post-conditioning)/(time spent staying in the I.N.P. box during the pre-conditioning)] was not significantly different among the groups. (**D**) Distances of locomotion in the I.N.P. box were significantly increased compared to those before receiving conditioning in the S3-6, S7-10, and S3-10 groups. Distances of locomotion after conditioning in the S3-6, S7-10, and S3-10 groups were significantly larger than the distance in the N3-10 group. Distance of locomotion after conditioning in the S3-6 group was significantly larger than the distances in the S7-10 and S3-10 groups. Distance of locomotion before conditioning in the S3-6 group was significantly larger than the distances in the N3-10 and S7-10 groups. (**E**) Preference ratios of locomotion distance. Preference ratios of locomotion calculated as [(locomotion distance in the I.N.P. box during the post-conditioning)/(locomotion distance in the I.N.P. box during the pre-conditioning)] were significantly higher in the S3-6, S7-10, and S3-10 groups than in the N3-10 group. †, *P* < 0.01; *, *P* < 0.05; a vs. b, *P* < 0.01; a vs. c, *P* < 0.05. Repeated measures two-way ANOVA (group × timing) followed by post-hoc Holm’s test.

**Figure 6:**
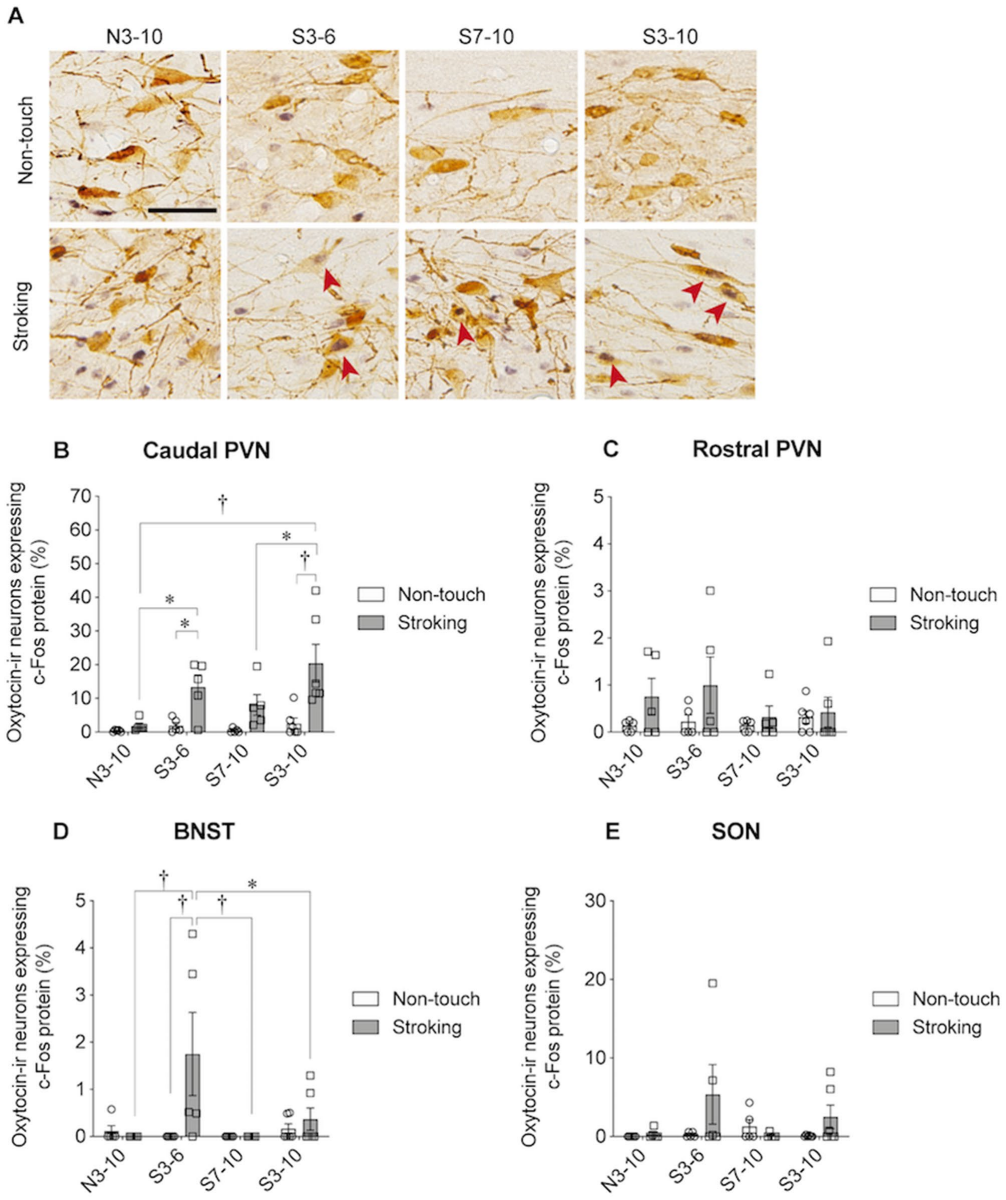
Results of immunohistochemical detection. (**A**) Photographs showing c-Fos immunoreactivity (black nuclear profiles) in oxytocin-ir neurons (brown cell body profiles). Expression of c-Fos protein in oxytocin-immunoreactive (-ir) neurons of the caudal hypothalamic paraventricular nucleus (PVN) following a non-touch condition or stroking condition in the N3-10, S3-6, S7-10, and S3-10 groups was examined. (**B**–**E**) Percentages of oxytocin-ir neurons expressing c-Fos protein in the caudal PVN (**B**), rostral PVN (**C**), bed nucleus of the stria terminalis (BNST) (**D**), and supraoptic nucleus (SON) (**E**). In the caudal PVN, the percentages of oxytocin-ir neurons expressing c-Fos protein were significantly higher after stroking in the S3-6 and S3-10 groups, but not in the N3-10 and S7-10 groups, than the percentage in non-stroking control rats. The percentages of oxytocin-ir cells expressing c-Fos protein following stroking stimuli were significantly higher in the S3-6 and S3-10 groups than in the N3-10 group. In the rostral PVN, the percentage of oxytocin-ir neurons expressing c-Fos protein was significantly higher after stroking than in the non-touch condition. In the BNST, the percentage of oxytocin-ir neurons expressing c-Fos protein was significantly higher after stroking in the S3-6 group than in non-stroking control rats. The percentage of oxytocin-ir cells expressing c-Fos protein following stroking stimuli was significantly higher in the S3-6 group than in the N3-10, S7-10, and S3-10 groups. In the SON, there was no significant difference. †, *P* < 0.01, *, *P* < 0.05. Two-way factorial ANOVA (group × stimuli) followed by post-hoc Holm’s test was performed. Error bars denote standard error of the mean. The red arrowheads indicate double-labeled neurons. Scale bar = 50 μm.

**Figure 7:**
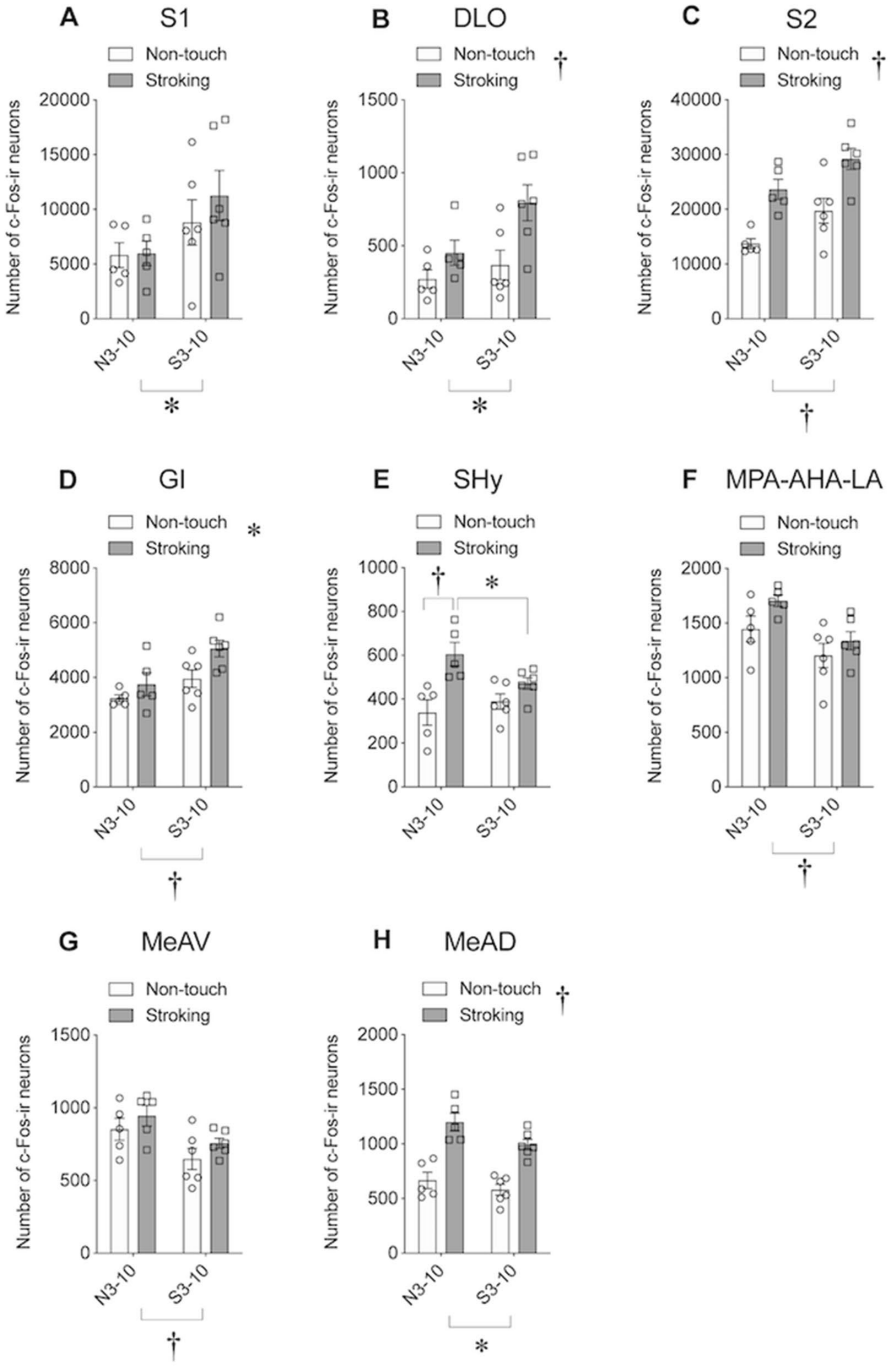
Number of c-Fos immunoreactive (-ir) cells after stroking stimuli. (**A**–**H**) Numbers of c-Fos-ir cells in the primary somatosensory cortex (S1) (**A**), dorsolateral orbital cortex (DLO) (**B**), secondary somatosensory cortex (S2) (**C**), granular insular cortex (GI) (**D**), septhohypothalamic nucleus (SHy) (**E**), medial preoptic area (MPA)-anterior hypothalamic area (AHA)-lateroanterior hypothalamic area (LA) (**F**), medial amygdaloid nucleus anteroventral part (MeAV) (**G**), and medial amygdaloid nucleus anterodorsal part (MeAD) (**H**). There was a significant effect of group (N3-10 vs. S3-10) in the S1, DLO, S2, GI, MPA-AHA-LA, MeAV, and MeAD. A significant effect of stimuli (non-touch vs. stroking) was also found in the DLO, S2, GI, and MeAD. There was significant interaction in the SHy. †, *P* < 0.01, *, *P* < 0.05. Two-way factorial ANOVA (group × stimuli) followed by post-hoc Holm’s test was used (see Supplementary Tables 1–3 for details).

**Figure 8:**
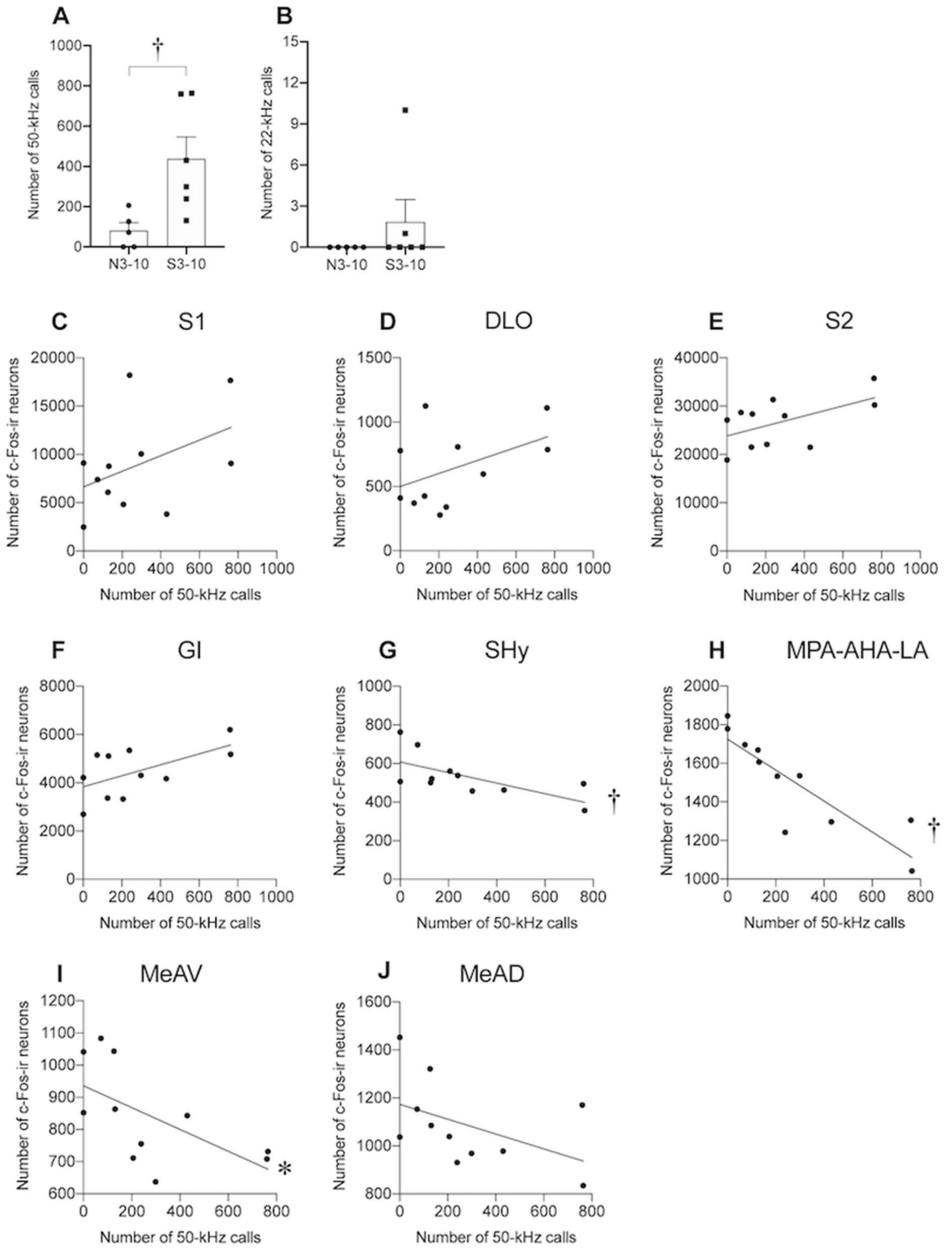
Number of ultrasonic vocalizations and correlation between number of c-Fos-ir cells and number of 50-kHz calls. (**A**,**B**) Numbers of 50-kHz calls (**A**) and 22-kHz calls (**B**) during stroking stimuli before brain sampling. (**C**–**J**) Correlations between numbers of c-Fos-ir neurons in the primary somatosensory cortex (S1) (**C**), dorsolateral orbital cortex (DLO) (**D**), secondary somatosensory cortex (S2) (**E**), granular insular cortex (GI) (**F**), septhohypothalamic nucleus (SHy) (**G**), medial preoptic area (MPA)-anterior hypothalamic area (AHA)-lateroanterior hypothalamic area (LA) (**H**), medial amygdaloid nucleus anteroventral part (MeAV) (**I**), and medial amygdaloid nucleus anterodorsal part (MeAD) (**J**) and number of 50-kHz calls during stroking stimuli. In the SHy, MPA-AHA-LA, and MeAV, there were significant negative correlations. †, *P* < 0.01, *, *P* < 0.05. Spearman’s correlation test.

Statistical analyses were performed using free software, HAD^51^ and R^52^. For one-way comparisons among multiple groups in which the variables have a normal distribution, one-way analysis of variance (ANOVA) was used. For analysis of data with a non-normal distribution, the Kruskal–Wallis test was used. For mixed model analysis of two-way layout data, two-way factorial or repeated measures ANOVA was used. The numbers of USVs at 3, 4, 5, 6, 7, 8, 9, and 10 weeks in the three stroking groups were analyzed separately for within-group comparisons by using Friedman’s test followed by post-hoc Holm’s test (Fig. 2 C–H). Data for the open field test (Fig. 3A) and preference score in the social preference test (Fig. 3B, right) were analyzed by one-way ANOVA followed by the post-hoc Holm’s test. Times spent in the bottle and hand areas in the social preference test (Fig. 3B left) were analyzed by repeated measures two-way ANOVA (group × area) followed by post-hoc Holm’s test. Times spent for following behavior in the following behavior test (Fig. 3C, left) were analyzed by one-way ANOVA followed by the post-hoc Holm’s test. The number of USVs during the following behavior test (Fig. 3C, right) was analyzed by the Kruskal–Wallis test followed by the post-hoc Holm’s test. Data for the numbers of 50-kHz and 22-kHz USVs in basal, holding, and stroking conditions (group × condition, Fig. 4A, B), numbers of vocalizations in FM and non-FM syllables (syllable pattern × condition, Fig. 4C–F), and times and locomotion distances in the CPP test (group × timing, Fig. 5B and D) were analyzed by repeated measures two-way ANOVA followed by post-hoc Holm’s test. Data for the preference ratio of the CPP test (Fig. 5C and E) were analyzed by one-way analysis of variance (ANOVA) followed by the post-hoc Holm’s test. Results of immunohistochemical experiments on oxytocin neurons (Fig. 6 and Supplementary Fig. 1) and c-Fos-ir neurons (Fig. 7A–H and Supplementary Fig. 2) were analyzed by two-way factorial ANOVA (group × stimuli) followed by post-hoc Holm’s test. The numbers of 50-kHz and 22-kHz calls in non-touch and stroking conditions (Fig. 8A, B) were analyzed by the Brunner-Munzel test.

Correlations between the number of c-Fos-ir neurons and number of 50-kHz calls in the N3-10 and S3-10 groups of rats that received stroking stimuli before brain sampling were analyzed by Spearman’s correlation test (N3-10 group, n = 5; S3-10 group, n = 6).

The percentages of animals in estrous stages (Supplementary Fig. 3) were analyzed by Fisher’s exact test^52^. *P* < 0.05 was considered statistically significant.

## Results

### Developmental changes of ultrasonic vocalizations

In the developmental period, 50-kHz and 20-kHz calls during stroking stimuli were recorded once per week (Fig. 2).

In the S3-6 group, rats emitted significantly larger numbers of 50-kHz calls during stroking at 5 or 6 weeks of age than at 3 or 4 weeks of age (Fig. 2C, P = 0.002, Friedman’s test, 3 vs. 5 weeks, *P* = 0.021; 3 vs. 6 weeks, *P* = 0.011, 4 vs. 6 weeks, *P* = 0.048, post-hoc Holm’s test).

In the S7-10 group, no significant difference was found among the numbers of calls at 7–10 weeks of age (Fig. 2E, P = 0.392, Friedman’s test).

In the S3-10 group, the number of 50-kHz calls increased as the age increased. The numbers of calls were larger at 6–10 weeks than at 3 or 4 weeks of age (Fig. 2G, P < 0.001, Friedman’s test, 3 vs. 6 weeks, *P* = 0.029; 3 vs. 7 weeks, *P* < 0.001; 3 vs. 8 weeks, *P* < 0.001; 3 vs. 9 weeks, *P* = 0.008; 3 vs. 10 weeks, *P* = 0.004; 4 vs. 7 weeks, *P* = 0.001; 4 vs. 8 weeks, *P* = 0.003; post-hoc Holm’s test).

The number of 22-kHz calls during stroking stimuli was not significantly different among ages within each group (Fig. 2D, F, H, Friedman’s test).

### Open field test

Anxiety-related behaviors were assessed by an open field test (Fig. 3A). No significant difference was found in total distances of locomotion (Fig. 3A left, *P* = 0.336, one-way ANOVA), total center times (Fig. 3A, middle, *P* = 0.603, one-way ANOVA), and immobile times (Fig. 3A right, *P* = 0.573, one-way ANOVA) among the groups.

### Social preference test

Preference to the hand of the experimenter (S.O.) as compared to a bottle was assessed by a social preference test (Fig. 3B). A significant area effect [F ^1,38^ = 248.146, *P* < 0.001], a significant group effect [F ^3,38^ = 20.804, *P* < 0.001], and a significant interaction [F ^3,38^ = 14.339, *P* < 0.001] were revealed by repeated measures two-way ANOVA. Rats in all groups stayed longer in the hand area than in the bottle area (Fig. 3B left, N3-10, *P* = 0.015; S3-6, *P* < 0.001; S7-10, *P* < 0.001; S3-10, *P* < 0.001, post-hoc Holm’s test). Rats in all three stroked groups stayed significantly longer in the hand area than did rats in the N3-10 group (N3-10 vs. S3-6, *P* < 0.001; N3-10 vs. S7-10, *P* < 0.001; N3-10 vs. S3-10, *P* < 0.001). In addition, rats in the S3-10 group stayed significantly longer in the hand area than did rats in the S3-6 and S7-10 groups (S3-6 vs. S3-10, *P* = 0.01; S7-10 vs. S3-10, *P* = 0.012, post-hoc Holm’s test).

Rats in the S3-6, S7-10 and S3-10 groups showed higher preference scores than rats in the N3-10 group did (Fig. 3B right, *P* < 0.001, one-way ANOVA, N3-10 vs. S3-6, *P* = 0.001; N3-10 vs. S7-10, *P* < 0.001; N3-10 vs. S3-10, *P* < 0.001, post-hoc Holm’s test).

### Following behavior test

Affiliative behavior toward the hand of the experimenter (S.O.) was assessed by a following behavior test (Fig. 3C). Rats in the S3-6 and S3-10 groups showed longer duration of following behavior toward the experimenter’s hand than rats in the N3-10 group did (Fig. 3C left, *P* < 0.001, one-way ANOVA; N3-10 vs. S3-6, *P* = 0.001; N3-10 vs. S3-10, *P* < 0.001, post-hoc Holm’s test). In addition, rats in the S3-10 group showed longer following behavior than rats in the S7-10 group did (*P* < 0.001, post-hoc Holm’s test).

Rats in the S3-10 group emitted a larger number of 50-kHz calls during the following behavior test than rats in the N3-10 group did (Fig. 3C right, χ^*2*^ = 14.273, *df* = 3, *P* = 0.003, Kruskal–Wallis test; *P* = 0.001, post-hoc Holm’s test).

### Ultrasonic vocalizations in basal, holding and stroking conditions

USVs were recorded in basal, holding and stroking conditions and categorized into 50-kHz calls and 22-kHz calls (Fig. 4).

In the number of 50-kHz calls, a significant group effect [F ^3,38^ = 4.245, *P* = 0.011], a significant condition effect [F ^2,76^ = 33.707, *P* < 0.001], and a significant interaction [F ^6,76^ = 3.397, *P* = 0.026] were found by repeated measures two-way ANOVA. In the S3-6, S7-10, and S3-10 groups, rats emitted significantly larger numbers of 50-kHz calls in the stroking condition than in the basal or holding condition (Fig. 4A, S3-6, basal vs. stroking, *P* = 0.001, holding vs. stroking, *P* = 0.002; S7-10, basal vs. stroking, *P* = 0.026, holding vs. stroking, *P* = 0.039; S3-10, basal vs. stroking, *P* < 0.001, holding vs. stroking, *P* < 0.001, post-hoc Holm’s test). In the S3-6, S7-10, and S3-10 groups, rats emitted significantly larger numbers of 50-kHz calls in the holding condition than in the basal condition (S3-6, *P* = 0.002; S7-10, *P* = 0.023; S3-10, *P* < 0.001, post-hoc Holm’s test). In the stroking condition, rats in the S3-6 and S3-10 groups emitted significantly larger numbers of 50-kHz calls than rats in the N3-10 group did (N3-10 vs. S3-6, *P* = 0.002; N3-10 vs. S3-10, *P* < 0.001, post-hoc Holm’s test). Rats in the S3-10 group emitted a larger number of 50-kHz calls in the stroking condition than rats in the S7-10 group did (*P* = 0.038, post-hoc Holm’s test).

On the other hand, no significant difference was found in the number of 22-kHz calls among the basal, holding, and stroking conditions [Fig. 4B, group effect, F ^3,38^ = 0.131, *P* = 0.941; condition effect, F ^2,76^ = 2.816, *P* = 0.093; interaction of the two, F ^6,76^ = 0.596, *P* = 0.653, repeated measures two-way ANOVA].

#### Syllable patterns of ultrasonic vocalizations

In the numbers of FM and non-FM calls in the N3-10 group, no significant effect of syllable pattern [F ^1,18^ = 0.27, *P* = 0.61], a significant condition effect [F ^2,36^ = 7.869, *P* = 0.01] and no significant interaction [F ^2,36^ = 0.739, *P* = 0.409] were found by repeated measures two-way ANOVA (Fig. 4C). Rats emitted a significantly larger number of USVs in the stroking condition than in the basal or holding condition (basal vs. stroking, *P* = 0.021; holding vs. stroking, *P* = 0.031, post-hoc Holm’s test). In addition, rats emitted a significantly larger number of USVs in the holding condition than in the basal condition (*P* = 0.029, post-hoc Holm’s test).

In the S3-6 group, no significant syllable pattern effect [F ^1,18^ = 2.313, *P* = 0.146], a significant condition effect [F ^2,36^ = 18.030, *P* < 0.001] and no significant interaction [F ^2,36^ = 2.794, *P* = 0.112, repeated measures two-way ANOVA] were found in the numbers of FM and non-FM calls (Fig. 4D). Rats emitted a significantly larger number of USVs in the stroking condition than in the basal or holding condition (basal vs. stroking, *P* = 0.001; holding vs. stroking, *P* = 0.001, post-hoc Holm’s test). In addition, rats emitted a significantly larger number of USVs in the holding condition than in the basal condition (*P* = 0.001, post-hoc Holm’s test).

In the S7-10 group, no significant syllable pattern effect [F ^1,18^ = 0.745, *P* = 0.4], a significant condition effect [F ^2,36^ = 8.55, *P* = 0.009] and no significant interaction [F ^2,36^ = 0.976, *P* = 0.336, repeated measures two-way ANOVA] were found in the numbers of FM and non-FM calls (Fig. 4E). Rats emitted a significantly larger number of USVs in the stroking condition than in the basal or holding condition (basal vs. stroking, *P* = 0.016; holding vs. stroking, *P* = 0.011, post-hoc Holm’s test). In addition, rats emitted a significantly larger number of USVs in the holding condition than in the basal condition (*P* = 0.023, post-hoc Holm’s test).

In the S3-10 group, no significant syllable pattern effect [F ^1,22^ = 4.287, *P* = 0.05], a significant condition effect [F ^2,44^ = 27.654, *P* < 0.001], and a significant interaction of the two [F ^2,44^ = 5.471, *P* = 0.028, repeated measures two-way ANOVA] were found in the numbers of FM and non-FM calls (Fig. 4F). Rats emitted a significantly larger number of FM calls in the stroking condition than in the basal or holding condition (basal vs. stroking, *P* < 0.001; holding vs. stroking, *P* < 0.001, post-hoc Holm’s test). Rats emitted a significantly larger number of FM calls in the holding condition than in the basal condition (*P* < 0.001, post-hoc Holm’s test). Rats emitted a significantly larger number of non-FM calls in the holding condition than in the basal condition (*P* = 0.001, post-hoc Holm’s test). In the stroking condition, rats emitted a significantly larger number of FM calls than the number of non-FM calls (*P* < 0.001, post-hoc Holm’s test).

### Conditional place preference (CPP) test

Rewarding value of stroking by the experimenter was assessed by a CPP test (Fig. 5). After conditioning with stroking stimuli, rats stayed longer in the I.N.P. box where they received stroking stimuli during conditioning as compared to rats before conditioning. Repeated measures two-way ANOVA showed no significant group effect [F ^3,38^ = 2.155, *P* = 0.109], a significant timing effect [F ^1,38^ = 54.028, *P* < 0.001], and no significant interaction [F ^3,38^ = 1.448, *P* = 0.244, repeated measures two-way ANOVA] (Fig. 5B). No significant difference was found in the preference ratio of time among the groups (Fig. 5C, P = 0.441, one-way ANOVA).

In the distances of locomotion in the I.N.P. box where rats received stroking during conditioning, a significant group effect [F ^3,38^ = 12.633, *P* < 0.001], a significant timing effect [F ^1,38^ = 128.614, *P* < 0.001] and a significant interaction of the two [F ^3,38^ = 8.896, *P* < 0.001, repeated measures two-way ANOVA] were found (Fig. 5D). Before conditioning, rats in the S3-6 group showed larger locomotion than rats in the N3-10 and S7-10 groups did (S3-6 vs. N3-10, *P* = 0.008; S3-6 vs. S7-10, *P* = 0.021, post-hoc Holm’s test). After conditioning, rats in the S3-6, S7-10, and S3-10 groups increased locomotion in the I.N.P. box as compared to rats before conditioning (before vs. after conditioning; S3-6, *P* < 0.001; S7-10, *P* < 0.001; S3-10, *P* < 0.001, post-hoc Holm’s test). Rats in the S3-6, S7-10, and S3-10 groups showed larger locomotion after conditioning than rats in the N3-10 group did (N3-10 vs. S3-6, *P* < 0.001; N3-10 vs. S7-10, *P* = 0.004; N3-10 vs. S3-10, *P* < 0.001, post-hoc Holm’s test). Rats in the S3-6 group showed larger locomotion after conditioning than rats in the S7-10 or S3-10 group did (S3-6 vs. S7-10, *P* < 0.001; S3-6 vs. S3-10, *P* = 0.021, post-hoc Holm’s test). Rats in the S3-6, S7-10, and S3-10 groups showed higher preference ratios of locomotion than rats in the N3-10 group did (Fig. 5E, P = 0.002, one-way ANOVA; N3-10 vs. S3-6, *P* = 0.013; N3-10 vs. S7-10, *P* = 0.012; N3-10 vs. S3-10, *P* = 0.003, post-hoc Holm’s test).

### Expression of c-Fos protein in oxytocin-immunoreactive (-ir) neurons

Expression of c-Fos protein in oxytocin-ir or non-oxytocin-ir neurons in the PVN, BNST and SON was examined in order to determine whether stroking stimuli activated these neurons (Fig. 6).

In the percentages of c-Fos-positive oxytocin-ir neurons of the caudal PVN, a significant group effect [F ^3,34^ = 4.928, *P* = 0.006], a significant stimuli effect [F ^1,34^ = 21.859, *P* < 0.001] and a significant interaction of the two [F ^3,34^ = 2.901, *P* = 0.049, two-way ANOVA] were found (Fig. 6B). In the S3-6 and S3-10 groups, but not in the N3-10 and S7-10 groups, the percentages of c-Fos-positive oxytocin-ir neurons following stroking were significantly higher than those in corresponding non-stroking control rats (S3-6, *P* = 0.01; S3-10, *P* < 0.001, post-hoc Holm’s test). The percentages of c-Fos-positive oxytocin-ir cells following the stroking stimuli were significantly higher in the S3-6 and S3-10 groups than in the N3-10 group (N3-10 vs. S3-6, *P* = 0.039; N3-10 vs. S3-10, *P* < 0.001, post-hoc Holm’s test). In addition, the percentage of c-Fos-positive oxytocin-ir cells following the stroking stimuli was significantly higher in the S3-10 group than in the S7-10 group (*P* = 0.021, post-hoc Holm’s test).

In the rostral PVN, no significant group effect [F ^3,34^ = 0.559, *P* = 0.646], no significant stimuli effect [F ^1,34^ = 4.133, *P* = 0.05], and no significant interaction of the two [F ^3,34^ = 0.637, *P* = 0.596, two-way ANOVA] were found in the percentages of c-Fos-positive oxytocin-ir neurons (Fig. 6C).

In the bed nucleus of the stria terminalis (BNST), a significant group effect [F ^3,34^ = 3.059, *P* = 0.041], a significant stimuli effect [F ^1,34^ = 4.209, *P* = 0.048] and a significant interaction of the two [F ^3,34^ = 3.636, *P* = 0.022, two-way ANOVA] were found in the percentages of c-Fos-positive oxytocin-ir cells (Fig. 6D). In the S3-6 group, but not in the other groups, the percentages of c-Fos-positive oxytocin-ir neurons following stroking stimuli were significantly higher than those in the corresponding non-stroking control rats (*P* = 0.001 in the S3-6 group, post-hoc Holm’s test). The percentage of c-Fos-positive oxytocin-ir cells following the stroking stimuli was significantly higher in the S3-6 group than in the N3-10, S7-10, and S3-10 groups (S3-6 vs. N3-10, *P* = 0.003; S3-6 vs. S7-10, *P* = 0.003; S3-6 vs. S3-10, *P* = 0.014, post-hoc Holm’s test).

In the supraoptic nucleus (SON), no significant group effect [F ^3,34^ = 1.185, *P* = 0.330], no significant stimuli effect [F ^1,34^ = 2.698, *P* = 0.110] and no significant interaction [F ^3,34^ = 1.716, *P* = 0.182, two-way ANOVA] were found in the percentages of c-Fos-positive oxytocin-ir cells (Fig. 6E).

No significant difference was found among the N3-10, S3-6, S7-10 and S3-10 groups in the numbers of oxytocin-ir cells in the caudal PVN, rostral PVN, BNST, and SON. In the numbers of oxytocin-ir cells in these four brain regions, no significant group effect, no significant stimuli effect, and no significant interaction were found [Supplementary Fig. 2, caudal PVN, no significant group effect, F ^3,34^ = 0.161, *P* = 0.922; no significant stimuli effect, F ^1,34^ = 0.097, *P* = 0.757; no significant interaction, F ^3,34^ = 0.241, *P* = 0.867; rostral PVN, no significant group effect, F ^3,34^ = 1.120, *P* = 0.355; no significant stimuli effect, F ^1,34^ = 0.034, *P* = 0.855; no significant interaction, F ^3,34^ = 0.228, *P* = 0.876; BNST, no significant group effect, F ^3,34^ = 1.162, *P* = 0.338; no significant stimuli effect, F ^1,34^ = 0.873, *P* = 0.357; no significant interaction, F ^3,34^ = 0.082, *P* = 0.970; SON, no significant group effect, F ^3,34^ = 0.973, *P* = 0.417; no significant stimuli effect, F ^1,34^ = 0.253, *P* = 0.618; no significant interaction, F ^3,34^ = 0.635, *P* = 0.598; two-way ANOVA].

The numbers of c-Fos-positive non-oxytocin-ir neurons in the BNST, rostral PVN and caudal PVN but not in the SON were significantly larger following stroking stimuli than before stroking. In the numbers of c-Fos-positive non-oxytocin-ir neurons in the BNST, rostral PVN and caudal PVN, but not in the SON, two-way ANOVA revealed no significant effect of group, a significant effect of stimuli and no significant interaction [Supplementary Fig. 2, BNST, no significant group effect, F ^3,34^ = 0.525, *P* = 0.668; significant stimuli effect, F ^1,34^ = 13.808, *P* = 0.001; no significant interaction, F ^3,34^ = 0.094, *P* = 0.963; rostral PVN, no significant group effect, F ^3,34^ = 0.421, *P* = 0.739; significant stimuli effect, F ^1,34^ = 24.241, *P* < 0.001; no significant interaction, F ^3,34^ = 0.051, *P* = 0.985; caudal PVN, no significant group effect, F ^3,34^ = 0.504, *P* = 0.682; significant stimuli effect, F ^1,34^ = 20.613, *P* < 0.001; no significant interaction, F ^3,34^ = 0.189, *P* = 0.903; SON, no significant group effect, F ^3,34^ = 0.742, *P* = 0.534; no significant stimuli effect, F ^1,34^ = 3.857, *P* = 0.058; no significant interaction, F ^3,34^ = 0.977, *P* = 0.415, two-way ANOVA].

### Expression of c-Fos protein in various brain regions

In order to identify brain regions activated during stroking stimuli and to explore brain regions in which activity was influenced by post-weaning stroking stimuli, expression of c-Fos protein was examined after stroking stimuli in various regions of the forebrain and midbrain in the N3-10 and S3-10 groups (Fig. 7).

In the number of c-Fos-ir cells, two-way fractal ANOVA analysis (group × stimuli) of brain regions observed in the present study showed significant effects of stimuli in various brain regions (see Supplementary Tables 1–3 for details). In contrast to the effects of stimuli, there were significant effects of groups in several regions: primary somatosensory cortex (S1) (Fig. 7A), dorsolateral orbital cortex (DLO) (Fig. 7B), secondary somatosensory cortex (S2) (Fig. 7C), granular insular cortex (GI) (Fig. 7D), medial preoptic area, anterior and lateroanterior hypothalamic area (MPA-AHA-LA) (Fig. 7F), and medial amygdaloid nucleus anteroventral part (MeAV) (Fig. 7G) and anterodorsal part (MeAD) (Fig. 7H). Significant interaction between group and stimuli was found only in the septohypothalamic nucleus (SHy) (Fig. 7E). Subsequent post-hoc analysis revealed that the number of c-Fos-ir neurons in the SHy was increased after stroking stimuli in the N3-10 group (Fig. 7E, P = 0.001, post-hoc Holm’s test). In addition, the number of c-Fos-ir neurons in the SHy following stroking stimuli was significantly larger in the N3-10 group than in the S3-10 group (Fig. 7E, P = 0.039, post-hoc Holm’s test).

### Correlation analysis

In order to clarify the brain regions in which activity was influenced by post-weaning stroking and was correlated with affiliative responsiveness to humans, we conducted correlation analysis as an exploratory investigation to line up candidates of brain regions responsible for affiliation development. We focused on brain regions where a group effect or interaction was found to be significant as shown in Fig. 7.

The number of 50-kHz calls during stroking before brain sampling in the S3-10 group was significantly larger than that in the N3-10 group (Fig. 8A, P < 0.001, Brunner-Munzel test). On the other hand, the number of 22-kHz calls was not significantly different between the S3-10 and N3-10 groups (Fig. 8B).

Among the brain regions where a significant group effect and significant interaction were found, the numbers of Fos-ir cells in the SHy, MPA-AHA-LA, and MeAV were negatively correlated with the number of 50-kHz calls during stroking stimuli before brain sampling (Fig. 8G, SHy , r = -0.743, *P* = 0.009; Fig. 8H, MPA-AHA-LA, r = -0.916, *P* < 0.001; Fig. 8I, MeAV, r = -0.729, *P* = 0.011, Spearman’s correlation analysis). In the S1 (Fig. 8C), DLO (Fig. 8D), S2 (Fig. 8E), GI (Fig. 8F), and MeAD (Fig. 8J), there was no significant correlation between the number of c-Fos-ir cells and the number of 50-kHz calls.

### Estrous cycle

The percentages of animals in each estrous stage did not significantly differ among the N3-10, S3-6, S7-10, and S3-10 groups (Supplementary Fig. 3).

## Discussion

In the present study, post-weaning stroking stimuli for more than 4 weeks facilitated the emission of 50-kHz calls, induced preference and affiliative behavior toward the experimenter’s hand, increased reward values in stroking stimuli, and induced activation of oxytocin neurons in the caudal PVN after stroking in female rats as reported in male rats. The present study further demonstrated that post-weaning stroking stimuli decreased the activity of several brain regions, particularly the SHy, and increased activity in cortical regions including the insular cortex. These findings suggested possible suppressive actions of the SHy and promotive action of some cortical regions in the affiliative relationship between humans and rats.

Massage-like stroking stimuli have been suggested to facilitate the development of an affiliative relationship between individuals^29,30^. Our previous study using male rats revealed that repeated application of stroking stimuli for more than 4 weeks induced positive emotion in response to stroking stimuli^28^. In the present study using female rats, post-weaning stroking stimuli induced emission of 50-kHz calls during the stroking condition as compared with basal and holding conditions. An 8-week period of stroking stimuli induced a larger number of FM calls than non-FM calls during the stroking condition, suggesting that intensive affiliative reactions were induced in response to stroking stimuli after long-term post-weaning stroking stimuli. The post-weaning stroking procedure also induced affiliative behavioral responses in female rats. The duration spent staying in the hand area was significantly longer than that in the novel object area in all four groups. The possible tendency for rats under an anxious condition to avoid novel objects might have contributed to the longer duration of staying in the hand area, especially in the control group (N3-10). However, the social preference score toward the experimenter’s hand was consistently higher in the S3-6, S7-10, and S3-10 groups than in the N3-10 group, suggesting that rats in the stroked groups showed preference toward the experimenter’s hand. It is tempting to speculate that stroked rats showed preference toward the experimenter’s hand as a result of rats recognizing the experimenter’s hand as a positive social signal. Further studies are needed to clarify whether preference of stroked rats toward humans is comparable to that toward salient conspecifics. The post-weaning stroked groups of rats showed increased locomotor activity in the box conditioned with stroking stimuli, suggesting that reward values of stroking stimuli were induced after repeated stroking procedures. These findings indicate that post-weaning stroking stimuli induce affiliative responsiveness in female rats as well as in male rats.

Steroid hormones affect various behaviors including social behavior^53^. However, in the current study, no significant bias was found in the distribution of estrous stages among groups before brain sampling, suggesting that the post-weaning stroking did not significantly bias the estrous cycles and being consistent with the idea that the difference in estrous stages was not the main cause of differences in the affiliative responsiveness in behavioral tests.

During domestication processes of wild animals, development of affiliative behavior toward humans has often been associated with reduced levels of anxiety^47,48^. Tickling during developmental periods has been reported to decrease anxiety-related behavior in rats in adulthood^54,55^. However, we previously found that post-weaning stroked groups of male rats show no significant difference in anxiety-related behavior^28^. Consistent with this finding, in the present study using female rats, there was no significant difference in anxiety-related behavior among the N3-10, S3-6, S7-10, and S3-10 groups. Further studies including investigation of other anxiety-related indexes including neuroendocrine responses need to be performed to clarify the relationship between affiliative behavior and anxiety-related behavior.

Our previous study showed that the first post-weaning 4-week period is important for induction of affiliative responses of male rats toward humans, although boundaries of the critical period are not clear. In the present study, all stroked groups of female rats showed higher preference to the experimenter’s hand and longer locomotor distance in the I.N.P. box in the CPP test than those in the N3-10 group. However, in the S7-10 group that received stroking stimuli during a later period of adolescence, the number of 50-kHz calls did not significantly change during the developmental period. There was no significant difference between the S7-10 group and non-stroked N3-10 group in the duration of the following behavior, number of 50-kHz calls during the stroking condition or percentage of oxytocin-ir neurons expressing c-Fos protein after stroking. These findings suggest that stroking in the early adolescent period is important for inducing an affiliative response in both sexes.

Oxytocin has been reported to be activated by massage-like stroking stimuli^28,35–37^ and to facilitate affiliative social behaviors^31–33^. Importantly, a recent study showed that activation of parvocellular oxytocin neurons promotes social interaction between intraspecific individuals of rats^56^. Parvocellular oxytocin neurons were shown to be mainly located in the caudal PVN^57–59^. All of those studies led us to propose the possibility that oxytocin neurons in the caudal PVN play an important role in the development and maintenance of the affiliative relationship between humans and rats in the same manner as that suggested for the development and maintenance of an intraspecific affiliative relationship^56^. Furthers studies such as experimental manipulations of activity of oxytocin neurons are needed to clarify the role of oxytocin neurons in the development of affiliative behavior toward humans.

In this study, we examined the numbers of c-Fos-ir neurons in various brain regions in the N3-10 and S3-10 groups, which showed differences in affiliative behavior. We examined brain regions that were suggested to contribute to social behavior, pleasant sensation, reward processing and negative emotion. Significant effects of group or significant interaction were found in 9 regions: S1, DLO, S2, GI, SHy, MPA-AHA-LA, MeAV, and MeAD. In the S1, DLO, S2, and GI, the numbers of c-Fos-ir neurons were significantly larger in the S3-10 group than in the N3-10 group. These findings suggested that post-weaning stroking procedures change the activity of neurons in these regions. In particular, the insular cortex may play an important role in responsiveness to gentle stroking stimuli because several studies have shown that gentle stroking stimuli that induce a pleasant sensation induced activity in the insular cortex in humans^60^. Knobloch and colleagues reported that PVN oxytocin neurons project their axons to the insular cortex of rats^34^. In addition, oxytocin receptors are expressed in the insular cortex^61^. Importantly, blocking of the role of oxytocin receptors in the insular cortex eliminated the approach behavior toward a conspecific^62^. From these studies, it is interesting to speculate that the insular cortex is downstream of hypothalamic oxytocin neurons and that activity of the insular cortex in the S3-10 group might have been related to processing of the pleasant sensation and affiliative motivation toward the experimenter.

In contrast to the cortexes, the numbers of c-Fos-ir neurons in the rostral hypothalamus and medial amygdala were decreased in the S3-10 group compared to those in the N3-10 group. The number of c-Fos-ir neurons in the SHy was increased after stroking stimuli only in the N3-10 group, and that number was significantly larger than the number in the S3-10 group. A previous study revealed that there were cholinergic fibers in the hypothalamic area including the SHy and MPA^63^, which have been shown to be activated in a stressful situation^64–66^. Brudzynski further revealed that microinjections of carbachol, a cholinergic agonist, into the anterior hypothalamic area and the vicinity of the septum could induce prolonged 22-kHz calls in rats^67,68^, suggesting that cholinergic inputs to the rostral hypothalamus are related to aversive negative emotion. In the present study, the numbers of c-Fos-ir neurons in the SHy, MPA, AHA, and LA showed negative correlations with the number of 50-kHz calls and the percentage of oxytocin-ir neurons expressing c-Fos protein in the caudal PVN. It is possible that suppression of activity of the SHy, MPA, AHA reduced negative emotion and contributed to affiliative behavior toward humans.

A previous study in which heterospecific bonding between lambs and a human was investigated showed that re-union with the caregiver who stroked lambs during the developmental period activated various brain regions including the olfactory bulb, medial amygdala and orbitofrontal cortex, suggesting involvement of a multisensory process in heterospecific bonding^7^. Activation of hypothalamic oxytocin neurons was also reported^7^. In the present study, stroking stimuli by the familiar experimenter increased the number of c-Fos-ir cells in the anterior olfactory area, medial amygdala, orbital cortex, insular cortex, cingulate cortex and hypothalamic oxytocin neurons. It remains to be determined whether the presence of the familiar experimenter itself as well as the combination of stroking stimuli and presence of the familiar experimenter activates these brain areas in rats. Further study will also be needed to examine whether common neural mechanisms are shared across species for interspecific affiliative relationships.

Our previous and present studies showed that post-weaning stroking induces 50-kHz calls both in male rats and female rats. However, there was a difference in the number of 50-kHz calls. The average number of 50-kHz calls emitted by the female rats in the S3-10 group during stroking was 370.8, while the average number of 50-kHz calls by male rats in the S3-10 group during stroking was 68.6 in our previous study^28^. Although these experiments were performed separately and thus the data cannot be compared directly, responsiveness to a gentle touch might differ between male and female rats. Panksepp and Burgdorf examined the effects of tickling stimuli and found that the number of 50-kHz calls in male rats was significantly larger than that in female rats^25^. Tickling mimics play-fighting behavior between rats, and males exhibit play-fighting behavior more frequently than females do^40,41^. Thus, differences in the number of 50-kHz calls during tickling might reflect sex difference in responsiveness to playing behavior. On the other hand, stroking stimuli mimic allo-grooming, which is often observed during the lactating period. We have no data about the sex differences in allo-grooming behavior between adult animals. Several studies have shown gender differences in pain perception^69^. Further studies will be needed to clarify sex differences in the responsiveness to non-noxious affective sensation including that induced by stroking and tickling.

In summary, our results indicate that post-weaning stroking, particularly in the early adolescent period, facilitates an affiliative response in female rats possibly via activation of oxytocin neurons in the caudal PVN. In addition, these affiliative rats showed increased neural activity in the cortexes including the insular cortex and decreased activity in the rostral hypothalamic areas and the SHy. These brain areas have been shown to contain oxytocin fibers and oxytocin receptors^34,59^. The insular cortex is involved in social affection via integrative sensory processing^62^, the medial amygdala is involved in the processing of odor and odor-induced fear response^70^, the sensory cortex is involved in pleasant sensation^71^ and the SHy and anterior hypothalamus are involved in negative emotion^63^. It is interesting to speculate that the activity of oxytocin neurons in caudal PVN might facilitate an affiliative relationship possibly by facilitating activity of the insular cortex and sensory cortex and by suppressing activity of the SHy, anterior hypothalamus and medial amygdala.

## Supplementary Information

Supplementary Information 1.

Supplementary Information 2.
