# Supplementary material for: Post-weaning stroking stimuli induce affiliative behavior toward humans and influence brain activity in female rats
Source: Sci Rep. 2021 Feb 15;11:3805. doi: 10.1038/s41598-021-83314-w (PMC7884793; doi:10.1038/s41598-021-83314-w)
Supplement: Supplementary file 1 — Supplementary Information 1. [file 41598_2021_83314_MOESM1_ESM.docx]

**Supplementary information**

Post-weaning stroking stimuli induce affiliative behavior toward humans and influence brain activity in female rats.

Shota Okabe, Yuki Takayanagi, Masahide Yoshida, Tatsushi Onaka^*^,

Supplementary Table 1

Supplementary Table 2

Supplementary Table 3

Supplementary Fig. 1

Supplementary Fig. 2

Supplementary Fig. 3

**Supplementary Table 1**

Results of comparison of the numbers of c-Fos-ir neurons in various brain regions (bregma 5.16. to 2.28 mm) between the N3-10 and S3-10 groups. *, *P* < 0.05. Two-way fractal ANOVA analysis (group × stimuli) followed by post-hoc Holm’s test.

**Supplementary Table 2**

Results of comparison of the numbers of c-Fos-ir neurons in various brain regions (bregma 0.48 to -2.92 mm) between the N3-10 and S3-10 groups. †, *P* < 0.01, *, *P* < 0.05. Two-way fractal ANOVA analysis (group × stimuli) followed by post-hoc Holm’s test.

**Supplementary Table 3**

Results of comparison of the numbers of c-Fos-ir neurons in various brain regions (bregma -5.4 to -8.04 mm) between the N3-10 and S3-10 groups. Two-way fractal ANOVA analysis (group × stimuli) followed by post-hoc Holm’s test.

**A**

**
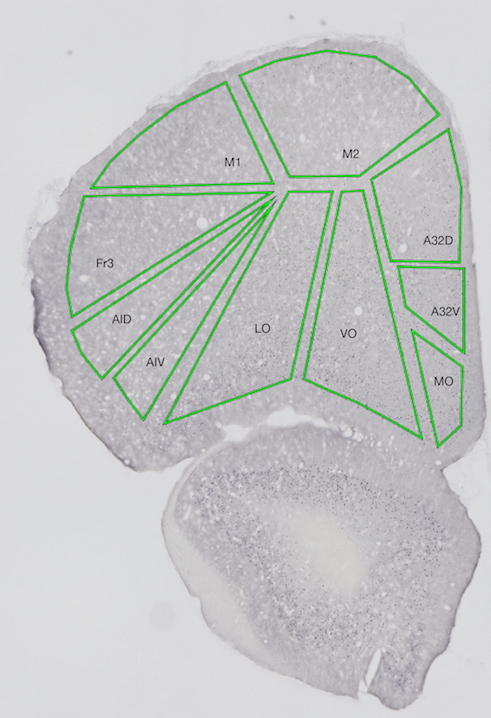
**

**B**

**
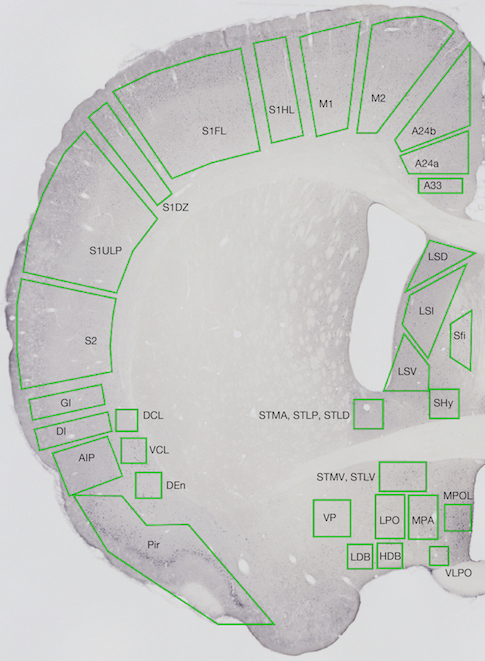
**

**C**

**
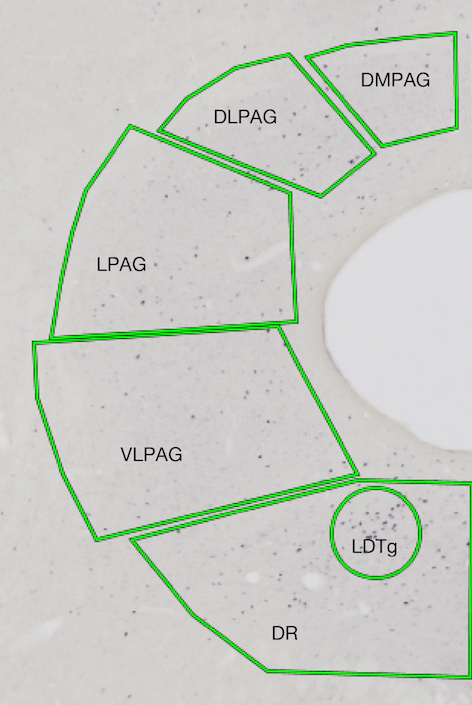
**

**Supplementary Fig. 1**

Representative example of regions of interest to examine c-Fos-ir neurons. Brain sections from 4.20 mm anterior to the bregma (A), from 0.12 mm posterior to the bregma (B), and from 8.04 mm posterior to the bregma (C).

**Supplementary Fig. 2**

Numbers of oxytocin-immunoreactive (-ir) neurons in the caudal PVN (A), rostral PVN (C), BNST (E), and SON (G). Numbers of c-Fos-ir neurons in non-oxytocin neurons in the caudal PVN (B), rostral PVN (D), BNST (F), and SON (H). The numbers of oxytocin-ir cells in the caudal PVN, rostral PVN, BNST, and SON were not significantly different among the four groups. The numbers of non-oxytocin-ir neurons expressing immunoreactivity of c-Fos protein were significantly increased after stroking stimuli in the caudal PVN, rostral PVN, and BNST but not in the SON. †, *P* < 0.01. Error bars denote standard error of the mean.

**Supplementary Fig. 3**

Percentages of animals in proestrus, estrus, metestrus, and diestrus. There were no significant differences (*P* = 0.475, Fisher’s exact test, (52)).
